# The formation of health-related procrastination in patients with type-2 diabetes: a grounded theory research

**DOI:** 10.3389/fpsyg.2023.1196717

**Published:** 2024-01-11

**Authors:** Habib Shareinia, Shahrzad Ghiyasvandian, Zahra Rooddehghan, Alireza Esteghamati

**Affiliations:** ^1^Department of Medical-Surgical Nursing, Faculty of Nursing, Gonabad University of Medical Sciences, Gonabad, Iran; ^2^Department of Medical-Surgical Nursing and Basic Sciences, School of Nursing & Midwifery, Tehran University of Medical Sciences, Tehran, Iran; ^3^Department of Internal Medicine, School of Medicine, Tehran University of Medical Sciences, Tehran, Iran

**Keywords:** health-related procrastination, type-2 diabetes, grounded theory, healthcare, family

## Abstract

Considering the adverse effects of procrastination on the care and treatment of patients with type-2 diabetes, examining the how, why, and contributing factors of procrastination in this group of patients through in-depth studies seems essential. This is a grounded theory research in which 28 participants were selected by theoretical purposive sampling from patients with type-2 diabetes, their families, and healthcare personnel. Data were collected through interviews, observations, field notes and memos. The data were analyzed using Corbin and Strauss approach (2015) and MAXQDA 2010 software. The data obtained were classified into seven main categories. Health-related procrastination in patients with type-2 diabetes began to form in the patients when they perceived the hardships of self-care as their main concern. The ominous shadow of the disease, the prison of family care, patient-related factors, health system shortfalls, sociocultural background, and the onslaught of the COVID-19 pandemic laid the groundwork for abandoning diabetes self-care. Patients tried to abandon their diabetes self-care by adopting strategies such as escaping the reality of the disease, self-medication and procrastination. Wavering health-related procrastination was identified as the central category of this research. The formation of health-related procrastination in patients with type-2 diabetes is a multidimensional process depending on the patients’ background that consists of their interactivity with their family, the health system, and the society. The findings of this research can be used for the design, implementation and monitoring of treatment and care programs for these patients.

## 1 Introduction

Demographic changes and cultural transition in societies and the aging populations have turned diabetes into a global epidemic. The International Diabetes Federation forecasts that 10.2% of the population will have diabetes in 2030 and 10.9% of the population in 2045 (Saeedi et al., 2019). The treatment and management of diabetes require a team approach in which the patient and their family are placed at the center as the team works to identify and solve the health-related needs of both the patient and their family (Ling et al., 2010). Even with significant efforts, keeping blood glucose levels normal remains out of reach, and finding solutions to confront the factors causing poor blood glucose control is not easy (Kasper et al., 2018).

In chronic diseases like type-2 diabetes, activities such as keeping a healthy diet, taking medications on time, self-care, stress management, and routine checkups play a key role in controlling disease symptoms and preventing its progress. These behaviors are potentially challenging and difficult due to factors such as fatigue, repetition, and other functional limitations associated with their performance. As a result, patients may resort to health-related procrastination and failure to perform health-related behaviors (Sirois, 2017).

Research findings suggest that procrastination in patients diagnosed with a chronic disease can seriously endanger their health and lead to earlier complications, higher treatment costs, and reduced quality of life. Increasing awareness about the dangers of procrastination both in patients with chronic diseases and among healthcare providers can potentially promote the health and quality of life of these patients (Sirois, 2016).

Health-related procrastination includes delays in performing health-related activities without valid reason and despite being able to perform these tasks and their accessibility and despite believing that they are beneficial and essential, and also awareness and fear of the negative outcomes of such delay (Kazemi et al., 2010; Steel, 2010; Rozental and Carlbring, 2014; Badri et al., 2016). Previous research suggests that procrastination is an important factor contributing to the poor management of chronic diseases such as cardiovascular disease and hypertension (Sirois, 2015). Azami-Aghdas et al. reported that procrastination is a key barrier to breast cancer screening (Azami-Aghdash et al., 2015). In a qualitative study, Rafiei et al. showed that procrastination is an important barrier to postpartum diabetes screening (Rafii et al., 2017). Research also suggests that people delay health-related tasks due to their being distressing (Golmohammadian et al., 2013), unpleasant (Kroese and De Ridder, 2016), difficult (Farzi et al., 2015), boring and tedious. Hence, procrastinators delay health-promoting behaviors such as seeking treatment and follow-up, which seem unpleasant and stressful (Golmohammadian et al., 2013).

Since procrastination is a style of behavior that postpones fulfilling tasks, and patients themselves are involved in forming such a behavior, it is a complex phenomenon and a variety of factors including social, cultural, economic, educational, family, health literacy, interactions of the individual with themselves and others, and the level of access to health services can affect it.

The present research aims to find models and processes that form procrastination in patients with type-2 diabetes in the context of social interactions, including in the family, hospitals and clinics, and adopts a comprehensive, precise, multidimensional approach to this phenomenon to identify these models and the links between them. Considering that health-related procrastination in patients with diabetes takes place in their interaction with other people over time, and since different categories of people, such as family members and healthcare providers, can become aware of the development of procrastination in a patient, and given that there is currently very little knowledge available on this subject, Corbin and Strauss grounded theory approach (2015) was used as an appropriate method to understand the process of health-related procrastination in patients with diabetes (Corbin and Strauss, 2015a). The present research thus investigates the formation process of health-related procrastination in patients with type-2 diabetes using the grounded theory approach.

## 2 Methods

### 2.1 Research design

Grounded theory is a qualitative research method seeking to discover processes and in which the researcher uses a systematic set of methods to inductively propose a theory about the studied phenomenon. With this method, a theory is inductively extracted from everyday experiences, interactions, documents, and observations. Grounded theory is process-oriented and helps us understand the changes that take place over time. Since sample size cannot be determined *a priori* in qualitative studies such as grounded theory and is contingent on data saturation (Corbin and Strauss, 2015a), purposive sampling was initially conducted in this research. Then, to select the next sample of participants, theoretical sampling was conducted until data saturation and development of the conceptual model. Health-related procrastination in patients with diabetes occurs over time and in social interactions with others; therefore, the grounded theory method was adopted to understand the process of health procrastination in patients with type 2 diabetes.

### 2.2 Sampling and participants

The participants were initially patients with type 2 diabetes. Then, interviews were conducted with nurses, physicians, other members of the healthcare team, and the patients’ companions, who had contact with these patients. The authors tried to ensure maximum variation in sampling (with respect to age, gender, education, duration of type 2 diabetes, or duration of receiving treatment). The first participants were purposively selected out of the patients with type 2 diabetes within the research population.

The participants, the patients’ family members, and the healthcare team members were selected based on the analysis of the data and theoretical sampling. During theoretical sampling, the selection of each individual depended on the data obtained from the previous participants. In fact, the researcher simultaneously collected, coded, and analyzed the data and decided what data to collect in the next steps and where to find them.

### 2.3 Data collection

After obtaining a code of ethics, the researcher visited the endocrinology and internal medicine wards and the diabetes clinic of Imam Khomeini Hospital Complex affiliated with Tehran University of Medical Sciences or any other location specified by the patient over the years (Xu et al., 2020). Purposive sampling was used to find the participants based on the inclusion criteria. The first participants of this research were patients with type-2 diabetes. In the next stage, interviews were also conducted with nurses, physicians, other healthcare personnel and the patients’ family. The patients’ family members and the healthcare personnel were selected according to the data analysis and theoretical sampling.

Patients diagnosed with type-2 diabetes were selected based on the inclusion criteria with the help of informants, including professors attending the diabetes clinic or the endocrinology and internal wards of the hospital and the nurses working in these wards. After visiting the clinic or inpatient wards to select the participants with the disease, the patients’ records and histories were studied and an initial brief interview was held with the patient to assess them based on the inclusion criteria. The inclusion criteria for the patients consisted of having the ability to establish good communication, willingness to participate in the study and be interviewed, speaking Persian, not suffering from acute mental illnesses, being under treatment for diabetes, and poor control of diabetes (HbA1c >8.5). The inclusion criteria for the healthcare team were the ability to communicate properly, willingness to participate in the study and being interviewed, speaking Farsi, and having at least 1 year of clinical work experience with patients with type 2 diabetes. As the research progressed and the intended categorizations of the theory gradually emerged, purposive sampling changed to theoretical type. The obtained data thus guided the researcher toward other people with maximum information required to complete the grounded theory approach, and this step continued until data saturation was reached. Written consent was obtained from all the participants, and they were ensured that they could withdraw from the research at any time and have all the relevant documents returned to them.

Participants who were introduced to the researcher by another person were checked for the inclusion criteria at the beginning of the interview, and were informed of who had introduced them. If they consented to participation, the interview continued.

The time and place of the interviews were selected in coordination with the participants. The interviews were held individually in a calm environment where they could feel at ease. Each interview took between 20 and 61 min depending on the circumstances and participant’s preference. The interviews with the participating patients were initially unstructured and asked general open-ended questions, such as: *How did you react when you were diagnosed with the disease? What decisions did you take? What did your family do for you?* A semi-structured interview then followed by asking questions including: *What do you do when you encounter a health-related problem? How do you manage diabetes-related problems?* Besides, to direct the interview and based on participants’ answers, other questions were also posed, such as: *What exactly do you mean? Please elaborate if possible. Please give an example so that I can understand you better.* At the end of the interviews, more open-ended questions were raised, such as: *Is there anything that you would like to add*?

With permission from the participants, the researcher recorded the interviews and took notes in the sessions whenever necessary. At the end of the interviews, the participants were told that another interview could ensue. Four of the participants (including three patients and one healthcare personnel) were interviewed twice in this research.

To ensure the credibility of the sampling process and the process of formation of health-related procrastination in patients with type-2 diabetes, three of the patients who had a long history of type-2 diabetes with controlled blood sugar (HbA1c <7) and had fully carried out all the diabetes-related treatment, care, and training activities were interviewed as negative cases. Observations and field notes taken in the research setting, such as the hospital wards and clinics where the interviews were held, and memos were used to develop the data obtained from the interviews, focus on the text, and help analyze and create the assumed categories.

### 2.4 Data analyses

This study used Corbin and Strauss theory (2015) for the data analysis, which includes five stages, namely open coding to understand the concepts, developing the concepts with regard to their features and dimensions, data analysis for the context, entering the process into analysis, and integration of the categories (Corbin and Strauss, 2015b). The researcher carefully listened to the recorded interviews several times and transcribed them verbatim in Microsoft Word before reading and reviewing them again. MAXQDA Ling et al. (2010) software was used for the data analysis. Given the messages conveyed by the words and sentences, the meanings were placed under them as codes for explanation. Then, the features and dimensions of the categories were determined and the patients’ main concern was established. An intention-outcome matrix was designed for the context data analysis. To take account of the process in the data analysis, the researcher sought answers to questions such as: *How do people react to the main concern* (i.e.*, hardships of self-care*)*?* The researcher wrote the storyline, reviewed the memos and drew diagrams to integrate the categories and find the central category.

### 2.5 Trustworthiness

Guba and Lincoln’s four criteria were used to ensure the validity of the qualitative data and the reliability of the results (Speziale et al., 2011). The researcher made efforts to increase the credibility of the findings and ensure the accuracy of the interpretations by contemplation and prolonged engagement with the research subject and by spending long hours in the research settings (attending the endocrinology and internal wards of the hospital and the diabetes clinic) and making consistent observations to get immersed in the data. To further improve the validity of the results, all the stages of the research were examined by experienced faculty members. The trustworthiness of the data was established by ensuring that the supervising professors’ comments were applied as an external control. To improve the confirmability of the research, the assumptions of the researcher were specified and recorded at the very beginning, faculty members supervised the research process, decision-making guidelines were set for the data collection and analysis steps, and all the important points during the research were noted down and continuously reviewed among the research team. To ensure the transferability of the results, participants’ demographic information was described to the extent that they would remain unidentifiable despite their demographic and cultural differences.

### 2.6 Ethical considerations

This research was approved by the Ethics Committee of Tehran University of Medical Sciences (IR.TUMS.FNM.REC.1398.207) and participants signed a research consent form.

## 3 Results

Of the 28 participants of this research, 16 were patients (10 males, 6 females), two were family members of the patients, and 10 were healthcare workers (three nurses, four physicians, one health policymaker, one nutritional therapist, and one psychologist). The age of the patients ranged from 40 to 69 years. The minimum duration of type-2 diabetes diagnosis was two and the maximum 25 years. The healthcare workers’ education ranged from undergraduate to subspecialty degrees (Table 1).

**Table 1:** Demographic details of the research participants.

Participant No.	Role	Gender	Age	Education	Disease duration/work experience (years)	Drugs used for diabetes	HbA1c	Experience or special features
1	Patient	M	67	Master’s degree	25	Oral	9.9	–
2	Patient	M	68	3rd grade of primary school	3	Oral, insulin (vial)	8.9	–
3	Patient	F	45	High school diploma	5	Oral, insulin (vial)	8.9	–
4	Patient	M	62	Bachelor’s	15	Insulin (vial)	9	–
5	Patient	M	63	5th grade of primary school	15	Oral, insulin (vial)	13	–
6	Patient	F	43	1st year of junior high	2	Oral, insulin (vial)	9	–
7	Patient	M	40	High school diploma	2	Oral, insulin (vial)	9.5	–
8	Patient	F	62	1st year of junior high	19	Oral, insulin (vial)	9.3	–
9	Patient	M	64	6th grade of primary school	4	Oral, insulin (pen)	12.6	–
10	Healthcare personnel	M	71	Endocrinology fellowship	40	–	–	–
11	Patient’s companion/relative	F	36	High school diploma	19	–	–	–
12	Healthcare personnel	F	51	MSN	27	–	–	–
13	Healthcare personnel	F	42	BSN	14	–	–	–
14	Healthcare personnel	F	40	BSN	16	–	–	–
15	Healthcare personnel	F	40	Endocrinology fellowship	7	–	–	–
16	Healthcare personnel	M	61	Endocrinology fellowship	30	–	–	–
17	Healthcare personnel	M	54	GP, MPH, health services management concentration	25	–	–	Diabetes manager at the Ministry of Health
18	Healthcare personnel	M	52	GP, diabetologist	22	–	–	Manager at the Iranian Diabetes Society
19	Patient	F	58	4th grade of primary school	9	Oral, insulin (pen)	8.8	–
20	Patient	M	54	1st year of junior high	20	Oral, insulin (pen)	8.8	–
21	Patient	M	48	3rd grade of primary school	17	Oral, insulin (pen)	13.2	–
22	Patient	F	41	2nd year of junior high	12	Oral, insulin (pen)	10.8	–
23	Healthcare personnel	M	37	MS in nutritional sciences	15	–	-	Work experience in diabetes training and diet
24	Healthcare personnel	M	42	PhD In psychology	18	-	-	Psychology group of the iranian diabetes society
25	Patient’s companion/relative	F	38	Bachelor’s degree	10	-	-	Diabetic foot ulcer, amputation
26	Patient	M	60	High school diploma	20	Oral	6.8	Negative case
27	Patient	M	69	Master’s degree	15	Oral	6	Negative case
28	Patient	F	63	Associate degree	23	Oral	6.5	Negative case

The research findings were classified into seven main categories for concept, six main categories for context, and two main categories for process (Table 2). The findings of the context data analysis showed that health-related procrastination in patients with type-2 diabetes is influenced by the multidimensional context of the hardships of self-care, which, by entering the process into the analysis, strategies including abandoning diabetes-related considerations (from escape to stubbornness and procrastination) were determined as a response to the hardships of self-care, which would eventually lead to impaired health.

**Table 2:** Formation concepts for health-related procrastination in patients with type-2 diabetes.

	Data analysis categories for the concepts	Main data categories for the context	Main data categories for the process	Central category
1	Multiple procrastination	Patient-related factors	From escape to stubbornness	Wavering health-related procrastination
2	Hardships of self-care	Shortfalls of the healthcare system in providing services	Procrastination	
3	Personality being inconsistent with self-care	The prison of family care		
4	Shortfalls of the healthcare system in providing services	Ominous shadow of the disease		
5	Unrealistic encounter with the disease	Sociocultural context		
6	Support against procrastination	Onslaught of the COVID-19 pandemic		
7	Normalization of the disease			

## 4 Data analysis for the concepts

### 4.1 Multiple procrastination

Patients with type-2 diabetes who participated in this research procrastinated in self-care, treatment, diabetic diet, medication administration, regular control of blood sugar, and physical activity.

A 54-year-old patient explained the reason for abandoning treatment and self-care as follows: *“When I get a little tired and fed up with these diets and medicines—which I should always take care to follow exactly—I just put all of them aside for a while. I tell myself to let go of the medicines, treatments, pills, or exercise. I ignore the doctors’ recommendations fully. But after a while, I realize that I’ve made a mistake.”* (*Participant* # *20*).

### 4.2 Hardships of self-care

Type-2 diabetes is a chronic disease for which patients suffer many pressures and restrictions in the process of self-care. Diabetes affects all aspects of life and makes adherence to the treatment and care recommendations a hard, exhausting, and complicated process.

A nurse said the following about the hardships of diabetes: *“Although there is honey [mellitus] in the name of this disease, it has a bitter taste and hurts the patients in every possible way. It’s as if patients become captive to it. After a while, they get fed up with how the treatments and care measures have affected their entire life.”* (*Participant* # *13*).

### 4.3 Personality inconsistent with self-care

Based on the research findings, personality affects how the patient controls diabetes. One of the participants explained their stubbornness in not following their treatment as: *“If someone gave me advice or instructed me to go to the doctor and treat myself, I’d get all worked up and hold a grudge against myself and say I will absolutely not go.”* (Participant #21).

A female participant who had lived with diabetes for over 20 years said that her strong will was one reason for her having a good control over the disease, adding: *“My personal will is one reason why I have a good control over my diabetes. I have a strong will in everything. I have promised myself to do everything I can to be healthy for as long as I live, so that I do not have to hold anyone prisoner to take care of me. This is very important to me.”* (Participant #28) (Negative case).

### 4.4 Shortfalls of the healthcare system in delivering services

One of the endocrinologists talked about the lack of telemedicine as a support system: *“We must have a support system for patients with diabetes, like telemedicine, that the patient can contact for training and to ask them questions. Under the present circumstances, such a support system is really wanting.”* (Participant #10).

A senior nutritionist who was interviewed in this study mentioned the lack of expert healthcare personnel and said: *“We have many patients with diabetes and their numbers grow every day. There aren’t enough doctors and nurses to cope with the patients, who do not have appropriate access to the healthcare workers.”* (Participant #23).

### 4.5 Unrealistic encounter with the disease

From what one of the patients said, it can be deduced that he is constantly escaping the truth of his disease: *“I’d go to the park and convince myself. I’d tell myself to never mind, to forget about the doctors and meds. Just exercise and you’ll be cured. Just exercise.”* (Participant #20).

The patient was a man who had suffered from long-term diabetes and had taken his medication regularly throughout these years. He said: *“I’ve become accustomed to my pills. I’ve been taking them for almost twenty years now. I do not mind taking pills at all. Putting a pill next to my plate of food and taking it is my duty and I must do it.”* (Participant #26) (Negative case).

### 4.6 Support against procrastination

Another endocrinology fellowship explained the importance of encouraging patients and said: *“An important task is to support the patients instead of criticizing them. People do not usually like being criticized. I tell the patients I know that living with diabetes is difficult. I train them in all aspects of life with diabetes and tell them that if they treat their disease properly they will have a good life.”* (Participant #16).

A female patient who had her husband’s support for and insistence on her taking the medications on time said: *“Many time, I’ve abandoned taking my medications and the doctors altogether. But my husband insisted by saying that I should go to the doctor, go to the lab for tests, go get my drugs. He would tell me to not keep postponing everything.”* (Participant #19).

### 4.7 Normalizing the disease

The analysis of the data showed that since diabetes is a lifelong disease, the patients’ focus on controlling their disease declines over time.

A clinical psychologist commented on how patients gradually procrastinate their disease control over time: *“I can say that most patients diagnosed with diabetes procrastinate their treatment and care in the long run.”* (Participant #24).

A patient who had diabetes for only 2 years was hurt by the lifelong nature of the disease and noted, “This disease is always with me. It never goes away or gets cured. It is with me for good. And all this going to the doctor will not cure it. Can it be healed? Will it go away? Of course not. So I’ve become quite used to what the doctors say, and I do not really care if I follow their recommendations or not.” (Participant # 7).

## 5 Main data analysis categories for the context

When the data analysis was completed, the main concern for patients was defined as the hardships of self-care. The hardships of self-care are influenced by underlying factors in the patient, their family, the nature of the disease, the healthcare system, COVID-19, the society, and the culture.

### 5.1 Patient-related factors

Patient-related factors, such as certain bad habits, beliefs, advanced age, and clinical status, create or exacerbate the hardships of self-care.

A patient discussed how his work preoccupations had reduced his adherence: *“Whenever I went on a work trip with colleagues, we were going to eat at a restaurant for 10, 15, or even 20 days that did not serve foods to my taste or suited for a diabetes regimen. This made me ignore physicians’ recommendations on my business trips. I told myself, ‘It’s OK. Forget about their advice for these few days. They’re impossible to follow here.’”* (*Participant* # *4*).

The interviewed nutritional therapist mentioned the difficulty of changing eating habits in older age: *“If someone has had the same diet for 60 or 70 years, if not longer, it is difficult for them to change their eating habits and diet now that they have been diagnosed with diabetes. This is a difficult task for them, and the older you are, the harder it gets.”* (Participant #23).

Another patient said the following about the effects of her mood on her care adherence: *“The doctor has written a prescription for me, but whenever I’m annoyed or upset, I just put the drugs aside. I tell myself, ‘Forget about it. I will not take it.’ If I get upset, I let go of everything.”* (*Participant* # *6*).

### 5.2 Shortfalls of the healthcare system in providing services

One of the participating nurses commented on nurses not spending enough time training the patients: *“I believe that training plays a big part in reducing procrastination in patients with diabetes. As nurses, we do not talk much with the patients and do not spend enough time with them to train them the way we should. We do not transfer our information to them.”* (Participant #14).

### 5.3 The prison of family care

The researcher found that certain family-related underlying factors pressurize patients with diabetes and discourage them from continuing with their treatment and self-care.

A 62-year-old woman who had lived with diabetes for 19 years was upset that her husband attended all her treatment visits: *“When my blood sugar goes up and I do not feel well, he does not let me go to the doctor [on my own]. He says we should go and come back together. When he wasn’t home, I did not dare go to the doctor on my own. If I want to go for blood work, I have to go with him. Well, when I see that I have to tell him about every visit so he can come along, I prefer not to go at all.”* (*Participant* # *8*).

A diabetologist explained the family’s over-support as follows: *“Sometimes, over-support from the family makes the patient with diabetes stubborn about not controlling their diabetes. We see this kind of over-support from children of older parents. For instance, the father wishes to leave the house, but his children will not allow it. They say, ‘If you go out, your blood sugar may drop. Remember last time when this happened when you went out?’”* (*Participant* # *18*).

### 5.4 The ominous shadow of the disease

Diabetes is a chronic disease that spreads its shadow over the patient’s life like a dark cloud, making its companionship notorious at all times in their life.

The interviewed fellowship of endocrinology commented on this issue and said: *“A person with diabetes must make several decisions to change their ways of living. Perhaps there is no other disease that can affect all the aspects of someone’s life to this extent.”* (Participant #16).

A patient who had been diagnosed with diabetes only 2 years ago commented on the chronic nature of this disease and said: *“This disease will always stay with me. The doctors say there is no cure for it, that it can just be controlled to stop from getting worse, but what good does that do? It will not get any better no matter how hard I try. It’ll stay with me for good.”* (Participant #7).

### 5.5 The sociocultural context

A participating nurse explained the influence of society and culture on care as follows: *“The culture of not caring for health means that everything is delayed and people do not take care of themselves. For instance, some patients say ‘we are too busy’, ‘I have a school-aged kid’, ‘I do not have enough time’. They come up with these excuses for not following their treatment and self-care. It is as if the culture and circumstances in which some people have grown up are so that health and treatment are not important to them at all.”* (Participant #13).

A diabetologist said the following about social support for patients with diabetes: “Psychosocial support plays a major role in diabetes treatment. Patients with diabetes must receive social support, but unfortunately, they receive little support. I believe that every patient with diabetes should be monitored by a psychologist too. Do we really have enough psychology clinics? Are there experienced therapists who have worked on diabetes at these clinics? (Participant # 18).

### 5.6 The onslaught of the COVID-19 pandemic

An older adult described his fear of presenting to treatment centers during the COVID-19 pandemic and stated: *“When COVID hit, I was afraid to come to the hospital. I came three times to get my medicines, but left and refused to be hospitalized. The last time they said your blood sugar will not drop with medications, you must be hospitalized; so I did get hospitalized in the end. I did not want to because I was afraid of COVID.”* (Participant #2).

## 6 Taking account of the process in the data analysis

At this stage, the analysis of the data showed that patients chose the solution of “abandoning diabetes-related considerations (from escape to stubbornness and procrastination)” to minimize the hardships of self-care.

### 6.1 From escape to stubbornness

According to the data analysis, the patients’ strategies in relation to this concept were “escape from the realities of the disease” and “self-medication.”

Initially, some patients were in denial of their disease, especially when they were first diagnosed. A patient who had elevated hemoglobin A1C levels said: *“When I get tired of going to the doctors’ so many times and taking all these drugs, I tell myself enough is enough. Am I even sick? No, forget it. Are we born into this world just to go to the doctors’ and take drugs? This is how I convince myself to not follow my treatment and care.”* (Participant #9).

One of the female patients talked about self-medication with herbal remedies: *“When I used the medicines from the local apothecary, I’d cut back on the medicines the doctor had given me, like, I would take only half the doses, or if the doctor told me to take three diabetes pills, I’d take only two and take the apothecary medicines instead.”* (Participant #19).

### 6.2 Procrastination

The analysis of the data in the present research showed that procrastination was the dominant solution adopted by the patients in response to the hardships of treatment and care.

An older adult patient described the lack of proper self-care as follows: *“I’m not a robot to do something at a set time. People sometimes abuse their own authority. I mean, they understand that they should not do something particular, but they do it nonetheless. Well, looking after your diet and taking your medicines is the same. A lot of the time, I do not abide by my diet and the time when I have to take my drugs.”* (Participant #4).

A patient with a long history of diabetes described how upset he was with consistently taking medications and said: *“Sometimes injecting insulin all the time and taking so many pills makes me angry. I say leave it. I do not want to take anything anymore –not insulin and not anything else. I simply do not care. I say to myself to let it go.”* (Participant #8).

## 7 Central category

Wavering health-related procrastination was a prominent variable in the data that was continuously evident in participants’ statements and covered all the categories and concepts with respect to their meaning and significance. The amount of health-related procrastination wavered in the patients like a yo-yo, depending on their circumstances and the underlying causes of this procrastination, but it could never be fully eliminated and kept oscillating like a pendulum. Oscillatory health-related procrastination led to mental harm, improper disease control, and costs incurred by patients, which eventually led to their “health deficit.”

An endocrine diseases professor explained the effects of patients’ procrastination on treatment: *“When patients say, ‘Let us live our lives and eat whatever we want now; come what may,’, we respond, ‘If you lose an eye, life will become more difficult. If you need dialysis, the treatment will be much harder for you and your family. There will be more mental suffering. If you continue procrastinating, the treatment will become more expensive, there will be more complications, and you’ll encounter further problems.”* (*Participant* # *16*).

A patient admitted to the endocrine diseases ward due to a diabetic foot ulcer explained the reason for the ulcer: *“I used to be careless, did not care about my disease, and would not listen to other people or the doctors. I did not know any better. But now, I see ulcers on my foot and should get an amputation.”* (*Participant* # *20*).

## 8 Discussion

**Figure 1:**
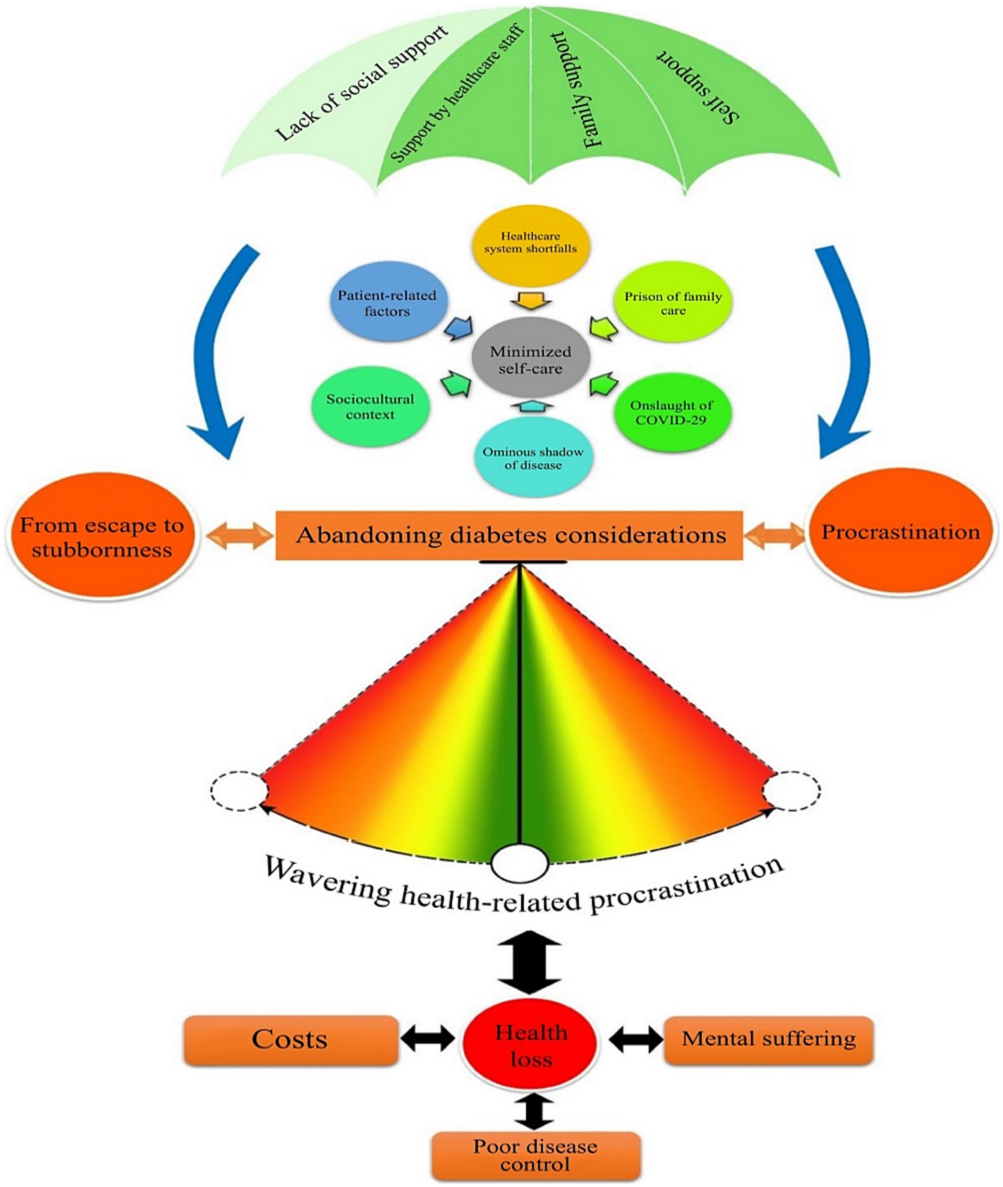
Conceptual model for the formation of health-related procrastination in patients with type-2 diabetes.

Based on the findings of this study, patients with diabetes procrastinated in several or all health-related dimensions, and their procrastination was more evident in self-care and treatment. They were often mindful of their health-related responsibilities, but procrastinated the implementation of decisions such as following up on their treatment and care, taking medicines on time, observing their diet, and engaging in physical activity (Figure 1).

Research shows that people who procrastinate their health-related tasks usually intend to carry them out, but procrastinate implementing their decisions (Stead et al., 2010; Rozental and Carlbring, 2014).

In a study on people with chronic diseases, Sirois found that procrastination is associated with stress, reduced health-promoting behaviors, and fewer regular medical and dental checkups (Sirois, 2016). A study by Rafiei et al. on postpartum diabetes screening showed that the participants intended to be screened but took no measures. They either avoided screening as a form of self-deception or decided that it had no immediate advantages. Procrastination was the most common reason for avoiding postpartum screening for diabetes (Rafii et al., 2017). These findings are consistent with the results of the present research suggesting that patients delay routine checkups such as laboratory tests and blood sugar monitoring.

In a qualitative study, Vluggen et al. found that following a healthy diet was difficult for patients and the temptation to eat unhealthy foods, stress, boredom, and social occasions and weekends may stop them from pursuing a diabetes diet. They also found that issues such as forgetting to take their medications, change of medications, and having to take medications at different times are responsible for the failure to regularly take blood sugar lowering medications (Vluggen et al., 2018). In a systematic review by Puvvada et al., the factors contributing to self-medication in patients diagnosed with type-2 diabetes included the low costs, ease of access, and fewer side-effects of traditional medicines (Puvvada et al., 2021). The participants of this research also attempted to control their diabetes with the arbitrary use of herbal medicines and drugs purchased from apothecary shops.

Haghbin and Pychyl examined the relationship between procrastination, physical activity, and a healthy diet. They found that people who have trouble with self-regulation and conscientiousness and are less satisfied with life follow an unhealthy diet and engage in less physical activity (Haghbin and Pychyl, 2016). In the current study, personality traits such as being disorganized, lack of self-control, and having a weak will were the basis of self-care bottlenecks and consequently health-related procrastination.

Murchison et al. investigated non-adherence to care for diabetes-induced ocular problems. The results of their retrospective study showed that patients with severe diabetic retinopathy were more likely than patients with mild diabetic retinopathy to make regular doctor’s visits and adhere to therapeutic and care-related instructions (Murchison et al., 2017). Nonetheless, in the present study, health-related procrastination was higher in patients with higher hemoglobin. This difference could be due to the acute nature and salient problems of retinopathy compared to the general complications of diabetes, which must have contributed to the faster and more regular follow-ups by patients with diabetic retinopathy.

The results of a study by Adu et al. on the barriers to self-management for patients with diabetes showed that disappointment due the chronic nature of diabetes, financial constraints, unrealistic expectations, and work- and environment-related factors restrict the effective management of diabetes in patients (Adu et al., 2019). The results of the present study showed that the extensive complications of diabetes, need to constantly adhere to the diet, and psychological pressures exerted by the family are some of the restrictions faced by patients with diabetes.

In their qualitative study on the challenges of diabetes management in Iran, Molayaghobi et al. stated that these challenges are system-oriented and patient-oriented and that the patient care system is weak and diabetes self-care is defective (Molayaghobi et al., 2019). Rushforth et al. identified the barriers to the effective management of primary care for type 2 diabetes by physicians and nurses as their limited knowledge and skills, high workload, large number of diabetes patients, negative feelings in dealing with diabetes, hopeless nature of the disease, and fear of complications of the treatment (Rushforth et al., 2016). Based on the results of the present study, the deficiencies of the healthcare system are among the factors that make patients abandon their diabetes-related considerations.

In another systematic review, Pamungkas et al. reported that family support positively affects patients with diabetes in adhering to their diet, efficiently managing the disease, improving their mental status and properly controlling their blood sugar (Pamungkas et al., 2017). In another study, Rasha Abd Elhameed et al. reported that married people who have the support of their family have better medication adherence than single people (Ali et al., 2021). The analysis of the data in this research showed that the support of the healthcare staff, the heartwarming support of the family, and self-support significantly contribute to reducing health-related procrastination in patients with diabetes.

Xu et al. showed that people with diabetes mellitus aged 60 years or above did not care for ensuring regular medication use and that medication non-adherence was higher in patients who had lived with diabetes for more than 5 years (Xu et al., 2020). Waari et al. also noted that patients who were diagnosed with diabetes for a longer time had less medication adherence (Waari et al., 2018). The results of our study also revealed that due to the chronic nature of the disease and the gradual development of its complications, patients’ attention to diabetes control gets diminished and they try to free themselves of diabetes-related considerations by adopting different strategies, such as procrastination or denying the truth of the disease.

In most chronic diseases, procrastination can harm the patient. Chronic diseases such as diabetes, which require regular and often daily management, or arthritis, which is sensitive to changes in stress levels, are good examples of a disease which may be negatively affected by procrastination in theory (Sirois, 2016). Studies show that non-adherence to proper self-care is an important factor contributing to diabetes complications and death. In fact, the successful control of diabetes depends on the level of patient self-care, because over 95% of the care required for diabetes is performed by the patients themselves (Anderson et al., 2000; Powell, 2008). Their results showed that patients’ procrastination, which was oscillatory and manifold, eventually led to deterioration of health, mental suffering, and increased costs with the poor control of diabetes, and there was a mutual and direct relationship between these factors.

### 8.1 Limitations

One of the limitations of this research was the old age of some patients and their physical problems, which interrupted and prolonged the interviews. Efforts were made to set up the interviews to suit the physical and mental conditions of the patients. The researcher tried to identify his presumptions and preconceptions on the research topic and write them down separately in order to prevent their interference with the study, since qualitative studies have a subjective nature and are affected by their researcher’s mentality.

## 9 Conclusion

Type-2 diabetes is a chronic disease requiring regular care and follow-up. The theoretical framework presented in this research showed that the process of health-related procrastination begins to take shape in patients with type-2 diabetes when they perceive the hardships of self-care as a main concern and then leads to their abandonment of diabetes-related considerations. Related contextual factors, such as the ominous shadow of the disease and the prison of family care, increase the patients’ inclination to abandon their health-related duties and exacerbate their pressures and obstacles, thus making the patient adopt multiple procrastination in their health-related duties. The ultimate outcome of health-related procrastination is impaired health, which leads to mental suffering for the patients and their families, poor disease control, and incurring costs. Understanding how health-related procrastination takes shape in patients diagnosed with type-2 diabetes can help focus on this issue when designing, implementing, and supervising treatment and care programs. Appropriate interventional strategies can then be designed to improve these patients’ treatment and care programs and to make them more tailored to the patients’ needs.

Considering the findings of the present research on the formation of health-related procrastination, establishing specialist centers such as polyclinics for diabetes will facilitate access to the relevant expertise, accelerate the disease investigation process and controlling complications, facilitate the use of educational services, guide patients with diabetes, raise their awareness levels, and change their attitudes and performance. Employing specialist nurses in diabetes is recommended to guide patients and increase synergy between the healthcare workers, patients, and their family members. Also, it appears necessary to familiarize healthcare workers with the concept of health-related procrastination in chronic diseases such as diabetes.

## Data availability statement

The raw data supporting the conclusions of this article will be made available by the authors, without undue reservation.

## Author contributions

HS, SG, and AE were involved in designing the study, organizing, and field supervision. HS, SG, and ZR were involved in writing and analyzing the results, reviewing and approving the final manuscript. All authors read and approved the final manuscript.
